# Everybody's Page

**Published:** 1888-07-07

**Authors:** 


### EVERYBODY'S PAGE.

It is said that two Dutch butter-making firms use for manufacturing adulterated or spurious butter no less than twenty thousand kilos of margarine daily.

The number of patients discharged cured in county and borough asylums during the ten years 1871-1880, was 40·30 per cent. on the admissions.

A child's chance of living is at its best about the age of twelve or thirteen. Then comes a change. The mortality after thirteen or thereabouts rises rapidly till about the twenty-fifth year, when there is a slight fall, and then a gradual rise till the end.

So far has the refinement of adulteration gone that a patent was actually taken out to protect a process which an ingenious inventor had elaborated for the manufacture of a certain mixture of chicory and coffee. This mixture was pressed into the shape of coffee berries, so that retailers of coffee might be fully prepared for those troublesome and fastidious patrons who, in order to make certain that they are being really supplied with pure coffee, insist on witnessing themselves the operation of grinding.

The Gotha Life Assurance Bank publishes some statistics of the mortality among its policyholders in 1887. From these it appears that the mortality of the medical members was 11·53 per cent. above the average of that of all the members taken together. The classes of disease to which this higher rate of mortality was due were diseases of the respiratory organs, phthisis, and infectious diseases—typhus fever having proved especially fatal to the younger members of the profession. There was only one case of post-mortem poisoning out of 1,052 deaths. Cerebral apoplexy was remarkably frequent, beginning as early as thirty-six years of age. There were fourteen cases of suicide, and four of accidental death. These are, however, below the general average.

The occurrence of paralysis might almost be looked upon as showing the benefits of civilisation, for it occurs only when civilisation has progressed sufficiently to allow to man a considerable duration of life. Perhaps the best view to take of it is to regard it as the normal mode of death, the fit termination of life, occurring however, at present, prematurely; and what science may expect to do, is not to eliminate it, as it hopes to do with consumption, heart diseases, fevers, &c., but to cause its onset to be postponed till about the hundredth year.

We are gravely assured, and it has been stated on oath by those interested in the sale of mustard, that it is altogether a mistake to use it pure. In such a condition we are told it won't keep, that its taste is not pleasant, and so on. A sufficient answer to all of this nonsense is that certain manufacturers make and sell really pure mustard, and that even if this could not be done, and that if mustard did require a little flour or some similar material to make it keep, it does not require for this purpose anything like the very large amount which is frequently put in. In cases of necessary admixture, if such cases there be, we should follow the example of a celebrated wit, when he astonished his milkman by appearing himself one morning on his doorstep, provided with two jugs, and politely requested to be furnished with the milk and water separately, as he intended henceforth to mix them himself.

Sir James Crichton Browne, M.D., in a report to the Education Department upon the alleged over-pressure of work in public elementary schools, informs us as the result of his investigations that the seeds of disease are being sown broadcast by the schoolmaster. We are led to infer from this report that water on the brain, various diseases of the kidney and heart, insanity, and suicide are increasing year by year, and that the main cause of this increase is over-pressure in elementary schools.

In tinned fruit there is frequently to be found a quantity of metallic impurity, arising no doubt from the juice of the fruit having dissolved some of the solder or even of the tin of the vessel in which the fruit was contained. This can hardly perhaps be called adulteration, and yet as the effects of eating such impure food might be very serious, it behoves the sellers of these articles either to assure themselves that their wares are free from poisonous matters or to withdraw them at once from sale.

Mr. A. Young, Edinburgh, has patented a hearing-trumpet. The bell of the trumpet is formed with the lower side slightly concave, the upper side being semicircular or arch-shaped. The bell passes backwards and is formed U shaped; the tubular part passes downwards and ends in a contracted part, in which a tube slides telescopically. A curved nipple, entering the ear, slides and swivels on the end of the tube. The bell is attached by its under surface to an elastic strip which terminates over the temples, thus holding the trumpet in position; a rear fastening, consisting of a spring-wire loop, is hidden in the hair or otherwise secured.

The old law of *noblesse oblige* seems to have lost its power, and to require reinforcement by law of a more vulgar order. This is the case in France at least, for the *Times* tells us that a Comte de Neuville has been arrested at Hyères charged with adulterating his wine. In a few weeks 250 persons at Hyères had suffered from giddiness and colic. The doctors pronounced the malady to be a sort of influenza, but there was one dissentient. Dr. Charles Roux traced it to the Neuville wine, and Marseilles, Toulon, and Hyères are ringing with expressions of indignation against the prisoner. Eleven bodies have since been exhumed of persons whose deaths are attributed to the drinking of the Comte de Neuville's wine.

At present the authorities have the power to prosecute anyone selling adulterated milk. But in order to get a conviction as the law at present stands, the case must be a very glaring one before the authorities are successful. Good milk should contain from 12 to 13 per cent. of solids. The Society of Analysts has fixed the percentage at 11·5, a very moderate standard indeed. In a prosecution, if the suspected milk barely comes up to this standard, the defendant can send a sample of the milk to Somerset House, and as the chemists there are the ultimate court of appeal as regards analysis, if they declare the milk pure the case breaks down. Now, the Somerset House authorities have never yet declared what is their standard; all that is known of it is that it is a very low one. For the ends of justice and for the sake of the public, it is time that this state of matters should cease, and that some recognised standard should be declared by the authorities, so that medical officers of health and analysts might have some grounds for recommending a prosecution. At present, under this most unsatisfactory arrangement, they are uncertain what to do except in a very bad case, and adulteration goes unpunished.

